# Analysis of clinical characteristics and treatment of immunoglobulin G4-associated cholangitis: A retrospective cohort study of 39 IAC patients

**DOI:** 10.1097/MD.0000000000009767

**Published:** 2018-02-23

**Authors:** Jianchun Xiao, Peiran Xu, Binglu Li, Tao Hong, Wei Liu, Xiaodong He, Chaoji Zheng, Yupei Zhao

**Affiliations:** Department of General Surgery, Peking Union Medical College Hospital, Chinese Academy of Medical Sciences, Beijing, China.

**Keywords:** clinical characteristics, IgG4-associated cholangitis, treatment

## Abstract

Immunoglobulin (Ig)G4-associated cholangitis (IAC) is one of the common organ manifestations of IgG4-related systemic disease (ISD). IAC and autoimmune pancreatitis (AIP) may mimic sclerosing cholangitis, cholangiocarcinoma, or pancreatic carcinoma. Diagnosis is based on a combination of clinical, biochemical, radiological, and histological findings.

To study the clinical presentation of and treatment strategy for IAC, we reviewed clinical, serologic, and imaging characteristics, as well as treatment response, in 39 patients with IAC. The majority of patients were men (82%). Clinical features on presentation included obstructive jaundice in 26 patients (67%) and abdominal pain in 20 (51%). Positive IgG4 immunostaining was seen in 27 patients. The median serum IgG4 level before treatment was 769.4 mg/dL (range, 309.1–1229.7 mg/dL). After the steroid therapy, the median serum IgG4 level in 23 patients was 247.0 mg/dL (range, 139.0–355.0 mg/dL). Cholangiograms were available in 36 (92%) patients. Stenosis of the lower part of the common bile duct was found in 26 of 39 patients. Stenosis was diffusely distributed in the intra- and extrahepatic bile ducts in 14 of 39 patients. Additionally, strictures of the bile duct were detected in the hilar hepatic lesions in 27 of 39 patients. AIP was the most frequent comorbidity (35/39 in this study) of IAC. Other affected organs included eyes (n = 6), salivary glands (sialadenitis, n = 10), lymph nodes (mediastinal and axillary, n = 3), kidneys (n = 2), and the retroperitoneum (retroperitoneal fibrosis, n = 2).

Regarding treatment, 29 patients were treated with steroids, of whom one underwent pancreatoduodenectomy, and one underwent choledochojejunostomy. Eight patients were treated with biliary stents. The remaining 19 patients took prednisolone alone. Eight patients achieved spontaneous resolution. Four patients with suspected pancreatic cancer or cholangiocarcinoma underwent surgery, including 2 patients who also received postoperative steroids. All patients were regularly followed up for 9 to 36 months. Only 2 patients in the steroids treatment group relapsed to manifest obstructive jaundice and high serum IgG4 levels. These 2 patients were treated with steroids and biliary stents, resulting in complete remission.

We also review the diagnostic and therapeutic management and discuss recent pathophysiological findings, which might aid in understanding the molecular mechanisms contributing to IAC and other manifestations of IgG4-related diseases (IgG4-RD). Biomarkers that are more accurate are needed to correctly diagnose IAC and prevent misdiagnoses and unnecessary therapeutic interventions.

## 1 Introduction

IgG4-related systemic disease (ISD) is a fibroinflammatory syndrome which involved multiple systems. It is characterized not only by the elevated levels of IgG4 but also multifocal IgG4-rich lymphoplasmacytic infiltration of involved organs.^[1]^ IgG4-associated cholangitis (IAC) is the biliary manifestation of ISD, and it presents with biliary strictures and obstructive jaundice. It was first reported by Björnsson et al^[2]^ in 2007.

IAC can present independently or together with other involved organs of ISD, especially autoimmune pancreatitis (AIP).^[3]^ IAC and AIP may be misdiagnosed as sclerosing cholangitis, pancreatic carcinoma, cholangiocarcinoma, or other biliary diseases. Doctors have made several diagnostic consensus criteria in the past decade, including the international consensus diagnostic criteria (ICDC),^[4]^ Japanese criteria,^[5]^ and the HISORt (histology, imaging, serology, other organ involvement, and response to therapy) criteria.^[3,6]^ Although immunosuppressive treatment shows excellent response of IAC, no accurate diagnostic test for IAC and ISD is available, often causing significant diagnostic delay. Therefore, more than a third of patients suffer from delayed diagnosis or misdiagnosis, even erroneous and extensive hepatobiliary surgery before they are confirmed as IAC.^[3,7]^ The pathophysiological mechanisms of IAC and other manifestations of ISD remain largely unknown.

We performed a retrospective observational study with the 39 IAC patients treated in our hospital with or without surgery. The aim of this study was to describe the characteristics of IAC, and the natural history after surgical and steroid therapy. Finally, we summarize our current understanding of the molecular and clinical features of this complicated biliary disease and discuss future perspectives.

## 2 Materials and methods

### 2.1 Study subjects

The study was approved by our institutional review board. Patients diagnosed with IAC (n = 39) between January 2012 and December 2016, who had multifocal intrahepatic/hilar strictures with or without common bile duct (CBD) involvement were included in this retrospective cohort study. The diagnosis was based on the HISORt criteria and Japanese criteria. Demographic data as well as clinical, laboratory, imaging, and histologic characteristics were assessed for all patients. In the patients treated with steroids, treatment response, and relapse rates were evaluated.

### 2.2 Histology and IgG4 immunostaining

IAC was diagnosed histologically from a resection specimen or core biopsy if there was a lymphoplasmacytic infiltrate within and around bile ducts with associated obliterative phlebitis and storiform fibrosis leading to sclerosis of the bile duct.^[8–10]^ The number of IgG4-positive plasma cells per high-power field (hpf) was counted in each specimen (Nikon E 600, field diameter 0.625 mm; Nikon, Tokyo, Japan).

### 2.3 Serology

IgG4 levels in serum (mg/dL) were measured in 23 patients who underwent steroid therapy using automated nephelometry^[11]^ (Behring Nephelometer II; Dade Behring, Inc., Newark, DE).

### 2.4 Imaging

Available computerized tomography, magnetic resonance imaging (MRI), and endoscopic retrograde cholangiograms were reviewed by radiologists. Strictures were characterized as distal (intrapancreatic), proximal extrahepatic, or intrahepatic.

### 2.5 Other organ involvement

The presence or history of retroperitoneal fibrosis, sialadenitis, mediastinal adenopathy, and kidney involvement was noted.

### 2.6 Response to treatment

Based on initial therapy at presentation, the patients were classified into 3 groups: surgical treatment (resections performed for suspicion of malignancy in this group), steroid therapy, and no treatment.

## 3 Results

### 3.1 Demographics and presenting clinical features

A total of 39 patients (32 men and 7 women) were diagnosed with IAC, with the criteria described in the introduction section. The majority of patients were men (82%). Clinical features on presentation included obstructive jaundice in 26 patients (67%) and abdominal pain in 20 patients (51%). (Table 1).

**Table 1:**
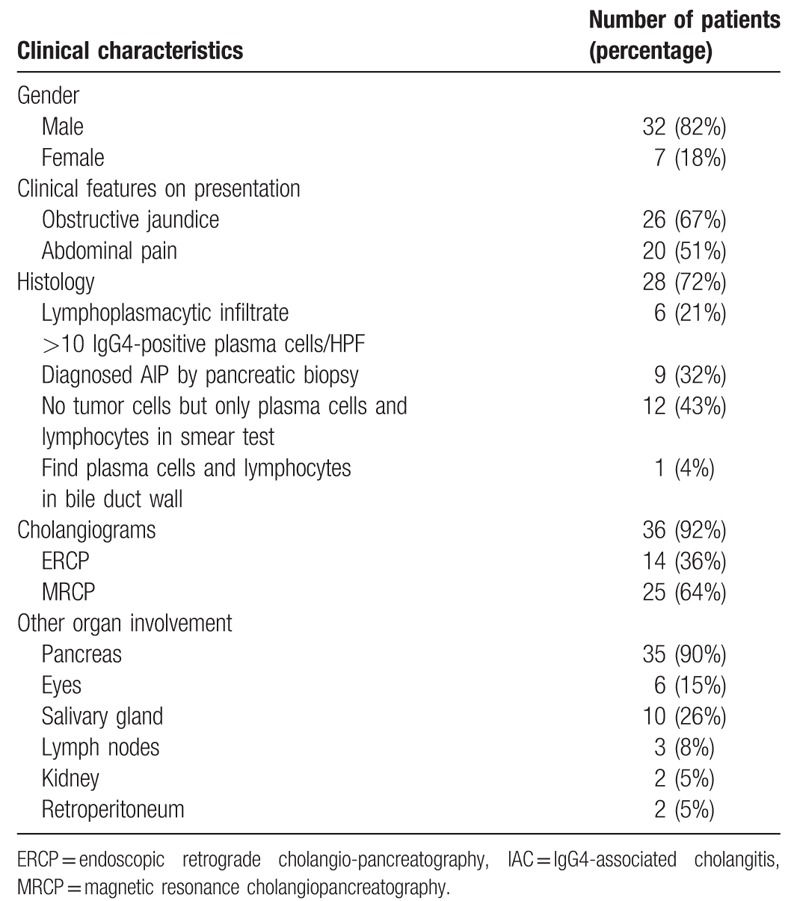
Clinical characteristic of 39 IAC patients.

### 3.2 Histology and IgG4 immunostaining

Biopsies were obtained for histology in 28 of 39 (72%) patients. A lymphoplasmacytic infiltrate associated with >10 IgG4-positive plasma cells per high-power field was demonstrated in 6 patients. Two patients were treated with pancreatoduodenectomy for the suspicion of pancreatic cancer, 1 patient was treated with choledochoplasty for suspected cholangiocarcinoma, 1 patient received choledochojejunostomy and cholecystectomy, and the remaining 2 patients had specimens taken from the esophagus and axillary lymph nodes. Nine patients were diagnosed with AIP through percutaneous pancreatic biopsy. Twelve patients exhibited only plasma cells and lymphocytes in smear test, with no tumor cells present. The remaining patient underwent a bile duct biopsy and infiltration of plasma cells was found, as well as lymphocytes in bile duct wall.

### 3.3 Serology

Serum IgG4 was measured during treatment and after completion of steroids in 23 patients (Table 2). The median serum IgG4 level before treatment was 769.4 mg/dL (range, 309.1–1229.7 mg/dL). All 23 patients had serum IgG4 levels of above the twofold cut-off value (280 mg/dL). After the steroid therapy, the median serum IgG4 level in these 23 patients was 247.0 mg/dL (range, 139.0–355.0 mg/dL). The serum IgG4 level in 8.7% of patients reached normal, and 65.2% of patients had levels below the twofold cut-off value.

**Table 2:**
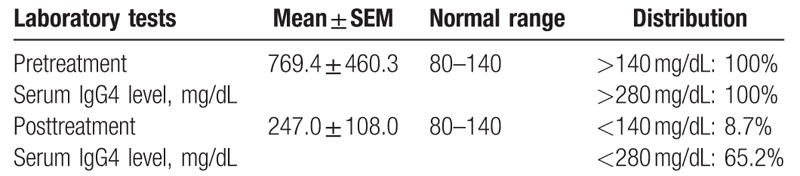
Pretreatment and posttreatment IgG4 level in 23 patients.

### 3.4 Imaging

Cholangiograms were available for 36 (92%) patients: 14 endoscopic retrograde cholangiograms and 25 magnetic resonance cholangiograms (3 patients had both). Stenosis of the lower part of the common bile duct was found in 26 of the 39 patients. Among them, 2 patients also had intrapancreatic biliary stenosis. Stenosis was diffusely distributed in the intra- and extrahepatic bile ducts in 14 of 39 patients. Additionally, strictures of the bile duct were detected in the hilar hepatic lesions in 27 of 39 patients (Table 3). Pancreas protocol computerized tomography scans or magnetic resonance cholangiopancreatography were available. A total of 20 patients had diffuse pancreatic enlargement.

**Table 3:**
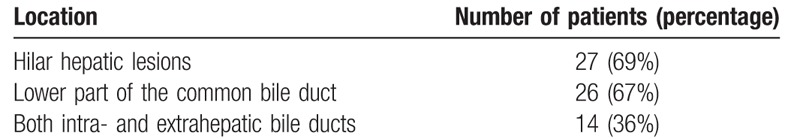
Location of common bile duct stenosis.

### 3.5 Other organ involvement

AIP was the most frequent comorbidity of IAC and the incidence reached 90% (35/39) in this study. Other affected organs included eyes (n = 6), salivary glands (sialadenitis, n = 10), lymph nodes (mediastinal and axillary, n = 3), kidneys (n = 2), and the retroperitoneum (retroperitoneal fibrosis, n = 2).

### 3.6 Response to treatment

A total of 29 patients were treated with steroids (initial prednisolone dose: 30–50 mg/d): 1 patient underwent a pancreatoduodenectomy, 1 patient underwent a choledochojejunostomy, 8 patients were treated with biliary stents, and 19 patients took the prednisolone alone. Prednisolone application resulted in a decrease in the IgG4 level in all 29 patients, but 2 of these 29 relapsed with manifesting obstructive jaundice and high levels of serum IgG4. These 2 patients were subsequently treated with steroids and biliary stents, and achieved complete remission. In the surgical group, all 4 patients responded to surgery, achieving resolution of strictures and/or normalization of liver enzyme levels. Eight patients had spontaneous resolution of strictures on initial presentation and did not receive treatment (Table 4). All patients were regularly followed up for 9 to 36 months. Baseline characteristics did not differ between the surgical and steroid-treated groups, with regard to IgG4 levels or stricture location.

**Table 4:**
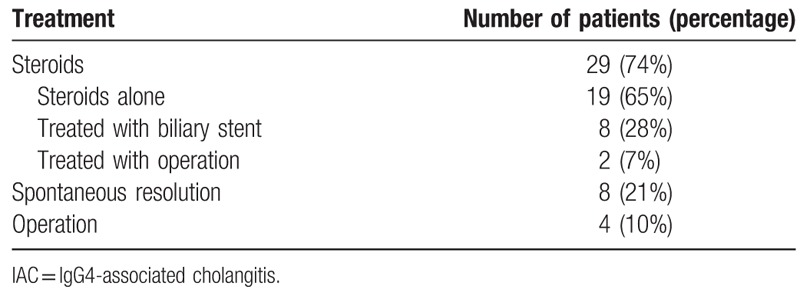
Treatment strategy of 39 IAC patients.

## 4 Discussion

Unlike typical autoimmune diseases, IAC more commonly happens in men, which are 4 times likely compared with women. Patients with IAC and AIP are usually over 60 years old.^[3]^ They present with weight loss, obstructive jaundice, or abdominal discomfort. Inflammation of the pancreas can also cause endocrine or exocrine insufficiency with diabetes and steatorrhea.^[7]^ Retroperitoneal fibrosis may lead to ureteral strictures which resulted in renal insufficiency.^[7]^ However, no specific symptoms enable reliable differentiation of IAC from other causes of biliary obstruction, leading to the serious consequences of misdiagnosis, which include surgical resection for presumed malignancy and inappropriate medical therapy.^[3,12]^ In our study, 82% of the patients were men, and obstructive jaundice and abdominal discomfort were present in more than half of them. Two patients underwent pancreatoduodenectomy as they were suspected to have pancreatic cancer.

Approximately 1 in 5 of patients had a history of allergic disorders such as bronchial asthma, drug allergies, or chronic sinusitis.^[13]^ A recent study showed that majority of patients with IAC and AIP in 2 independent cohorts were blue-collar workers which had long-term exposure to organic solvents, industrial gases, and other harmful agents.^[14,15]^ These findings suggest that chronic exposure to chemicals and toxins might be critical in the development of the disease.

Making the diagnosis of ISD is challenging as there is no effective test with a high accuracy available. The disease is named after the elevated serum concentrations of IgG4 (sIgG4). A previous study confirmed that about 80% of patients with ISC had elevated IgG4 by using a specific cut-off value of 135 or 140 mg/dL.^[1,16]^ In our study, the serum IgG4 levels of all 23 patients were above the twofold cut-off value. Furthermore, increased serum IgG4 levels are also observed in 5% to 25% of inflammatory, autoimmune, and malignant pathologies, and in 5% of healthy individuals.^[1,17,18]^ A serum IgG4 level >560 mg/dL increases the specificity and positive predictive value to 100% for differentiating IgG4-SC from PSC and cholangiocarcinoma.^[1,19]^ The other way is to calculate the ratio of IgG4 to IgG1 or total IgG.^[16]^ A ratio cut-off line such as IgG4/IgG >0.10 or IgG4/IgG1 >0.24 is statistically significant when doctors distinguish ISC from other biliary diseases.^[16,19,20]^ A further study suggested that serum IgG4 levels >180 mg/dL and >210 mg/dL gave a specificity of 97% and 100%, respectively, to distinguish type 3 (distal and hilar strictures) and type 4 (hilar stricture) IgG4-SC from cholangiocarcinoma.^[21]^

IAC patients also present with other serological abnormalities such as IgG elevations, antinuclear antibodies (ANAs), rheumatoid factor, and hyper-c-globulinemia.^[16,22]^

There are some characteristic image findings in computed tomography and MRI that are useful for the diagnosis of IAC, including multifocal biliary strictures, a smooth outer margin, a markedly thickened wall of bile duct, or a narrow but visible lumen.^[23]^ And we will find hyperenhancement during the late arterial phase, homogeneous hyperenhancement during the delayed phase, concurrent gallbladder wall thickening, and lack of vascular invasion.^[23–27]^

Tissue acquisition to enable accurate pathological diagnosis is a priority in IAC. IAC has 5 histological patterns: evident portal inflammation (with or without interface hepatitis); large bile duct obstructive features; portal sclerosis; lobular hepatitis; and canalicular cholestasis in perivenular areas.^[28]^ Immunostaining for IgG4 reveals the massive infiltration of IgG4-positive plasma cells. For surgical specimens, the cut-off values for IgG4-positive plasma cells are >50 cells/hpf. As for biopsy samples, the cut-off values are >10 cells/hpf.^[16,29]^

In the early days, many patients of IAC underwent surgery because of potential malignancies, indicating the primary differential diagnosis of IAC should be cholangiocarcinoma.^[16]^ In this field, bile duct biopsy and biliary cytology could do better than liver needle biopsy.^[16,30]^ If the imaging result shows no improvement after 2 to 3 weeks of prednisone treatment, the doctors should reevaluate the patients’ condition and make sure giving the right diagnosis.^[16]^ PSC has different clinical features; for example, it is likely in patients who are under 40 years old and those who have IBD.^[16]^ Furthermore, if we can get the specimens of bile duct, PSC has more damaged mucosa tissue with ulceration and xanthogranulomatous inflammation.^[16]^

Unlike European studies, AIP and IAC may coexist in most cases reported on many Asian cohorts.^[31]^ In our study, 35 patients (90%) had AIP, of the 4 patients who did not have AIP, 2 patients were diagnosed with IAC successfully, 1 was misdiagnosed with cholangiocarcinoma, and the other was misdiagnosed with pancreatic cancer. Differential diagnosis between cholangiocarcinoma and IAC is challenging due to significant overlap of clinical manifestations, lab tests, and imaging characteristics. Given the age, sex, clinical, and imaging characteristics of those patients, carcinoma was the primary probable diagnosis. Endoscopic retrograde cholangiopancreatography might fail to examine the whole bile duct due to the full-length stenosis, so diagnostic laparotomy was performed.

The regular initial treatment for both IAC and AIP is prednisolone at time of diagnosis for at least 3 months.^[15]^ Different starting doses are used all over the world. Patients with IAC are at high risk of relapse, with the majority occurring within 6 months of discontinuing or tapering steroid treatment.^[32]^ Known risk factors for relapse include the presence of proximal bile duct strictures and increased IgG4 levels.^[33]^ Compared with no maintenance treatment, several studies showed a reduction in relapse rates during long-term treatment with low-dose steroids or immunomodulators.^[15,34,35]^ Rituximab, a CD20-depletion agent, has been used in patients with IAC with incomplete remission, steroid dependency, or steroid or immunomodulator intolerance.^[33,36]^ However, rituximab has some serious adverse events, including cardiac arrest, severe infusion reactions, and reactivation of viral infections, not to mention the high costs. Doctors should take the escape therapy into account with reservation and only use it for well-defined cases which are insensitive to steroid treatment.^[15]^

In summary, we reviewed the clinical data of 39 IAC patients treated in our hospital. In order to avoid unnecessary surgery or misdiagnosis, it is critical to develop accurate diagnostic markers, but the initial trigger that sets off the aberrant IgG4 formation is still uncertain. In this study, only 39 IAC patients in a single center were retrospectively recruited. More studies are needed to attain a better understanding of its pathophysiology.
